# Molecular profiling of beer wort fermentation diversity across natural *Saccharomyces eubayanus* isolates

**DOI:** 10.1111/1751-7915.13545

**Published:** 2020-02-25

**Authors:** Wladimir Mardones, Carlos A. Villarroel, Kristoffer Krogerus, Sebastian M. Tapia, Kamila Urbina, Christian I. Oporto, Samuel O’Donnell, Romain Minebois, Roberto Nespolo, Gilles Fischer, Amparo Querol, Brian Gibson, Francisco A. Cubillos

**Affiliations:** ^1^ Facultad de Química y Biología Departamento de Biología Universidad de Santiago de Chile Santiago 9170022 Chile; ^2^ Millennium Institute for Integrative Biology (iBio) Santiago 7500574 Chile; ^3^ VTT Technical Research Centre of Finland Ltd VTT FI‐02044 Espoo Finland; ^4^ Laboratory of Computational and Quantitative Biology CNRS Institut de Biologie Paris‐Seine Sorbonne Université F‐75005 Paris France; ^5^ Departamento de Biotecnología de los Alimentos Grupo de Biología de Sistemas en Levaduras de Interés Biotecnológico Instituto de Agroquímica y Tecnología de los Alimentos (IATA)‐CSIC E‐46980 Valencia Spain; ^6^ Institute of Environmental and Evolutionary Science Universidad Austral de Chile 5110566 Valdivia Chile; ^7^ Center of Applied Ecology and Sustainability (CAPES) Pontificia Universidad Católica de Chile Santiago Chile

## Abstract

The utilization of *S. eubayanus* has recently become a topic of interest due to the novel organoleptic properties imparted to beer. However, the utilization of *S. eubayanus* in brewing requires the comprehension of the mechanisms that underlie fermentative differences generated from its natural genetic variability. Here, we evaluated fermentation performance and volatile compound production in ten genetically distinct *S. eubayanus* strains in a brewing fermentative context. The evaluated strains showed a broad phenotypic spectrum, some of them exhibiting a high fermentation capacity and high levels of volatile esters and/or higher alcohols. Subsequently, we obtained molecular profiles by generating ‘end‐to‐end’ genome assemblies, as well as metabolome and transcriptome profiling of two Patagonian isolates exhibiting significant differences in beer aroma profiles. These strains showed clear differences in concentrations of intracellular metabolites, including amino acids, such as valine, leucine and isoleucine, likely impacting the production of 2‐methylpropanol and 3‐methylbutanol. These differences in the production of volatile compounds are attributed to gene expression variation, where the most profound differentiation is attributed to genes involved in assimilatory sulfate reduction, which in turn validates phenotypic differences in H_2_S production. This study lays a solid foundation for future research to improve fermentation performance and select strains for new lager styles based on aroma and metabolic profiles.

## Graphical abstract

This article demonstrates the extensive molecular differences between *S. eubayanus* strains underlying volatile compound production during fermentation.

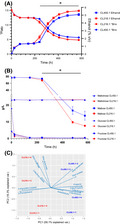

## Introduction

The choice of a yeast strain in brewing depends on myriad factors, including fermentation performance, ethanol and hop tolerance, sugar consumption profile and production of volatile compounds (e.g. acetate esters, ethyl esters and higher alcohols) (Gibson, *et al.*, 2017). In recent years, the phylogenetic relationships and phenotypic diversity of ale brewing strains – mostly *Saccharomyces cerevisiae* – have been studied in depth, with a focus on fermentation performance and the genetic mechanisms responsible for expressed phenotypes (Gallone, *et al.*, 2016; Gibson, *et al.*, 2017; Mendes, *et al.*, 2017). In contrast, lager yeasts – denoted *Saccharomyces pastorianus* – have not been studied to the same extent. Lager yeasts are hybrids between *S. cerevisiae* and the cold‐tolerant *Saccharomyces eubayanus* (Gibson, *et al.*, 2017); however, the natural history of these yeasts remains obscure (Baker, *et al.*, 2015). The first isolation of *S. eubayanus* in South America in 2011 was particularly puzzling given the Central European roots of lager beer (Libkind, *et al.*, 2011). Since then, this species has however been found in New Zealand, Canada, United States, East Asia and Chile (Bing, *et al.*, 2014; Gayevskiy and Goddard, 2016; Peris, *et al.*, 2016; Nespolo, *et al.*, 2019), demonstrating its presence in cold regions across the globe. In fact, *S. eubayanus* can efficiently grow at a range of lower temperatures (4–25°C) compared with *S. cerevisiae*, providing unique opportunities for brewing (Mertens, *et al.*, 2015). The differences in cold tolerance may be linked to mitochondrial DNA differences (Baker, *et al.*, 2019).

The recent isolation of *S. eubayanus* from Argentinian Patagonia (Libkind, *et al.*, 2011; Eizaguirre, *et al.*, 2018) is a promising step towards utilizing a new set of strains for brewing, either directly or through the generation of new *Saccharomyces* hybrids from crosses with *S. cerevisiae* (Krogerus, *et al.*, 2015; Gallone, *et al.*, 2019; Langdon, *et al.*, 2019). However, only few studies have addressed the fermentative capacity in *S. eubayanus*, mostly due to the reduced number of available strains, being only recently explored in cider (Magalhaes, *et al.*, 2017), wine (Alonso‐Del‐Real, *et al.*, 2017) and beer (Krogerus et al., 2017a,2017b; Eizaguirre, *et al.*, 2018; Nespolo, *et al.*, 2019). The challenge is evident as shown by recent studies demonstrating genetic differences across *S. eubayanus* populations (Peris, *et al.*, 2016; Eizaguirre, *et al.*, 2018; Nespolo, *et al.*, 2019), potentially indicative of a large phenotypic diversity among all isolates. Nevertheless, there are no detailed reports of the *S. eubayanus* brewing potential in terms of fermentation capacity, metabolite production and aroma profiling in genetically distinct individuals.

Molecular studies aiming to understand the transcriptional and molecular wiring of *S. eubayanus* strains are restricted to the impact of carbon source assimilation and gene regulation in the presence of maltose, and maltotriose in *S. cerevisiae* x *S. eubayanus* hybrids (Brickwedde, et al., Brouwers, *et al.*, 2019). Thus, the understanding of simple and complex traits in *S. eubayanus* is not only limited by the lack of available isolates, but also by the absence of large‐scale assays addressing their biology and physiology under fermentative conditions. Here, we present the fermentative profiles of eight *S. eubayanus* strains collected in Chile together with the CBS12357^T^ (Argentina) and the P1C1 isolates (New Zealand). Based on large differences in wort fermentative profiles, two strains were selected for further analysis and their genomes fully sequenced and assembled using nanopore technology. Moreover, the fermentation profile of the two strains was characterized in depth through metabolome, transcriptome and volatile compound production, providing detailed evidence of the genetics underlying their phenotypic and fermentative differences.

## Results

### Extensive fermentation profile differences across strains

**Figure 1:**
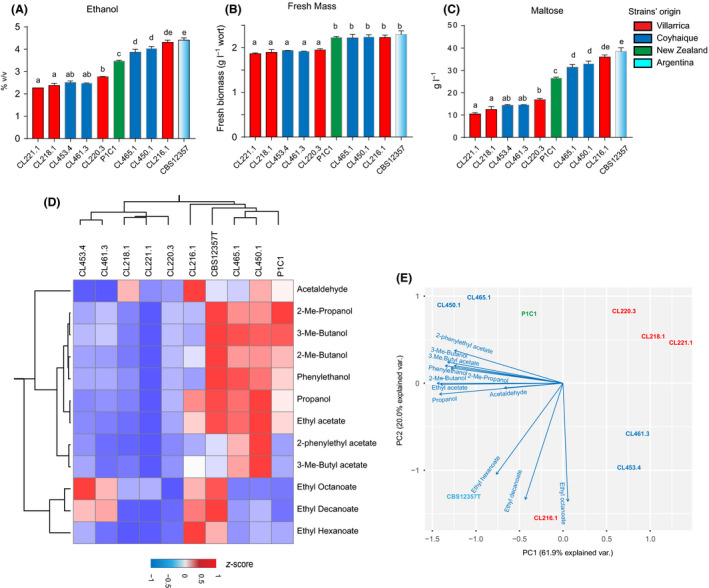
Metabolite levels in fermented wort by native *S. eubayanus* strains. A. Ethanol production, (B), fresh mass and (C) sugar consumption were evaluated in Patagonian strains, including the type strain (CBS12357^T^) and the New Zealand isolate P1C1. Maltose is represented as consumption of the total amount initially found in wort. Similarly, (D) volatile compound production of interest in beer by native *S. eubayanus* strains was obtained. *Z*‐scores are shown (E) principal component analysis (PCA) of volatile compound production across strains. Fermentations were performed in duplicates (*n* = 2) for 168 h, and a one‐way ANOVA test was performed to evaluate for statistically significant differences between strains. Letters depict significant differences between strains.

In order to determine whether wild *S. eubayanus* strains isolated from the Villarrica (middle Patagonia) and Coyhaique (South Patagonia) regions exhibited differences in brewing performance, we performed a fermentation round in 15 °P wort utilizing a set of ten isolates – including as references the CBS12357^T^ (Libkind, *et al.*, 2011) and the P1C1 (Gayevskiy and Goddard, 2016) strains, isolated from Argentina and New Zealand respectively. After seven 168 h of fermentation, we measured ethanol production, total fresh mass, residual maltotriose, maltose, glucose and fructose (Table S1, Fig. 1). Strains exhibited significant differences in ethanol production (*P*‐value < 0.05, ANOVA, results to be taken with caution *n* = 2) with levels ranging from 2.38 ± 0.01 (mean ± standard error) v/v to 4.31 ± 0.07 v/v in the CL218.1 and CL216.1 strains, respectively, both from Villarrica (Fig. 1A). Similarly, significant differences were found for total fresh mass (Fig. 1B, Table S1) and maltose consumption (Fig. 1C), where strains with the greatest maltose consumption produced higher ethanol and fresh biomass levels. No differences were found for glucose and fructose consumption, as both sugars were fully consumed by all strains, or maltotriose which was not utilized by any *S. eubayanus* strains (Table S1). The type strain CBS12357^T^ produced the greatest ethanol levels and consumed the highest levels of maltose across all strains (Fig. 1). Overall, when strains were grouped based on their geographic origin, no significant differences were found between both localities (Villarrica and Coyhaique, *P*‐value > 0.05, ANOVA), and only significant differences were found between individual strains, independently of the locality where the strain was obtained from.

We quantified the production of volatile compounds in this set of strains by measuring the concentration of twelve metabolites related with aroma and off‐flavours produced in beer fermentation. The metabolites included were esters, higher alcohols and carbonyl compounds in the final fermented wort (see methods and Table S2 for a full list of compounds). For all compounds, we found significant differences between strains (Table S2). Interestingly, some strains showed high levels of desirable aroma‐active metabolites, such as 3‐methylbutyl acetate, an ester which provides a banana‐like aroma. Production levels were high with CL450.1 compared with the other strains. Similarly, other strains such as CL216.1 revealed high ethyl ester production, with relatively high levels of ethyl hexanoate and ethyl decanoate potentially providing apple and fruity aromas (Fig. 1D).

To determine common metabolite regulation and the volatile compound production profile for each strain, we performed a principal component analysis (PCA) utilizing the twelve phenotype scores (Fig. 1E). The first two components explain an overall variance of 61.9% (PC1) and 20% (PC2) and are grouped by fermentation performance (PC1) and the chemical nature of the compounds detected, either ethyl esters (bottom PC2 axis) or higher alcohols (top PC2 axis, Fig. 1E). Based on this distribution, some strains grouped closely to ethyl esters, while others to higher alcohols. For example, strains CL465.1 and CL450.1, both from Coyhaique, grouped next to higher alcohols and acetate esters like 2‐phenylethyl acetate and 3‐methyl butyl acetate, while CL216.1 and the type strain grouped next to ethyl esters. The other strains did not show clear tendencies and did not cluster near any group of compounds, likely influenced by their weak fermentation performance and the production of intermediate/low levels of all metabolites. Furthermore, hierarchical clustering of all isolates based on metabolite production was also used to group strains (Fig. 1D). In this case, strains were grouped by the chemical family of the compounds preferentially produced, and not by their geographic origin, suggesting that genetic variants underlying volatile compound production between strains are present in both populations and do not segregate between isolation sites. Our results demonstrate the extensive diversity in terms of volatile compound production across *S. eubayanus* strains.

### Fermentation performance differs between strains in a brewery environment

**Figure 2:**
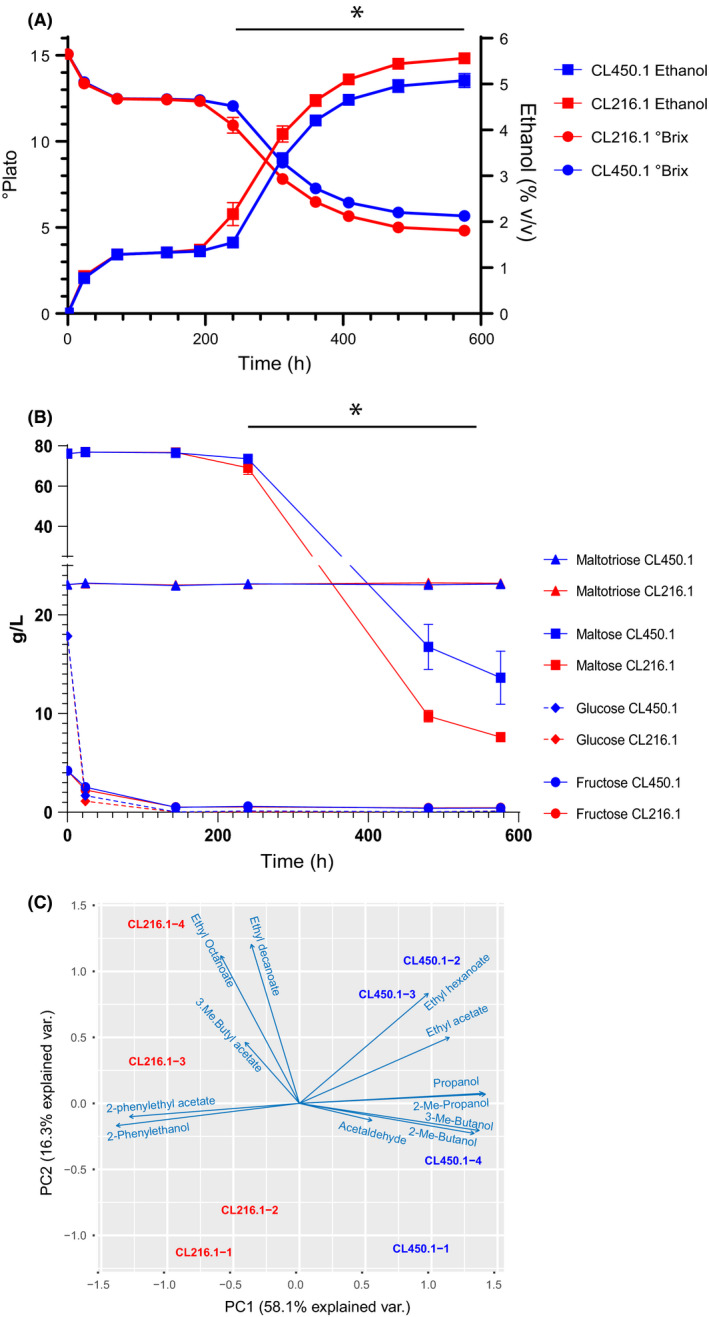
Fermentation profile of CL216.1 and CL450.1 in semi‐industrial brewing conditions. A. Attenuation (°Plato) and ethanol production (%v/v) were evaluated during 576 h (24 days). B. Glucose, fructose, maltose and maltotriose consumption was measured across the fermentation (C) A PCA of volatile compound production across the two strains. Fermentations were performed in quadruplicate (*n* = 4), and a one‐way ANOVA test was performed to evaluate for statistically significant differences between strains. (*) denotes time points exhibiting significant differences between strains.

In order to fully characterize the fermentative profiles of the two strains producing the most ethanol in small‐scale experiments (Villarrica CL216.1 from PB‐3 lineage and Coyhaique CL450.1 from PB‐1 lineage, Fig. 1A), fermentations were carried out under experimental settings mimicking industrial conditions. For this, fermentations were performed in quadruplicates in 2 l vessels with 15 °Plato all‐malt wort at a temperature of 15°C, and samples were taken throughout the process. Ethanol production and residual sugar curves showed the occurrence of a pause phase, likely due to the switch between different carbon sources (Fig. 2A). The total amount of ethanol produced started to significantly differ between the two strains after 240 h of fermentation and by the end of the fermentation reached 5.6 ± 0.04 v/v and 5.1 ± 0.15 for CL216.1and CL450.1 respectively (Table S3, *P*‐value < 0.05 ANOVA), in agreement with the small‐scale experiments (Fig. 1A). Sugar consumption was measured throughout the fermentation process for fructose, glucose, maltose and maltotriose (Fig. 2B and Table S3). Glucose was rapidly depleted from the media after 24 h, and no maltose or maltotriose consumption was detected at this time point (Fig. 2B). Maltose consumption was only detected after 240 h, in agreement with a glucose repression effect that delays the switch between the utilization of glucose and other carbon sources. Interestingly, maltose consumption significantly differed between strains at the end of the fermentation process (*P*‐value < 0.02, ANOVA), with CL216.1 consuming on average 87% of the maltose, compared with CL450.1 which was able to consume 78% of this carbon source. The difference in the ability of both strains to consume maltose probably explains their variable ethanol production. Overall, after 480 h in the 2 l tank systems, both strains consumed high levels of maltose and produced high levels of ethanol, yet glucose repression significantly impacted fermentation time. These results demonstrate and validate the fermentative differences in greater volumes between *S. eubayanus* strains from different populations and demonstrate their potential utilization for large fermentation tanks under industrial conditions.

We also determined the production of 12 volatile compounds after 144, 480 and 576 h of fermentation in both strains. Overall, statistically significant differences were found for seven and five compounds at the middle (144 h) and end of fermentations (480 h) respectively (Table S3). For example, the Coyhaique CL450.1 strain showed lower 2‐phenylethanol production at both time points compared with the CL216.1 Villarrica strain, while the opposite was obtained at the end of the fermentation for 3‐methyl butyl acetate. Moreover, comparison of the whole volatile compound production profiles demonstrated significant differences at the end of the fermentation (*P*‐value < 0.04, paired *t*‐test), but not during mid‐fermentation (*P*‐value = 0.8, paired *t*‐test, Table S3), demonstrating that once most sugars have been consumed, both strains have distinctive aromatic profiles in the final beer. A PCA using all four replicates per strain by day 20 explained 74.4% of the variation in the first two dimensions (Fig. 2C). The first dimension separated the replicates based on their genotype and compounds preferentially produced, where compounds grouped according to their chemical family. In this way, our results demonstrate that CL216.1 showed a better fermentation profile and the production of greater levels of volatile esters.

### Metabolomic analysis under fermentative conditions reveals differences in the assimilation of carbon and nitrogen sources

**Figure 3:**
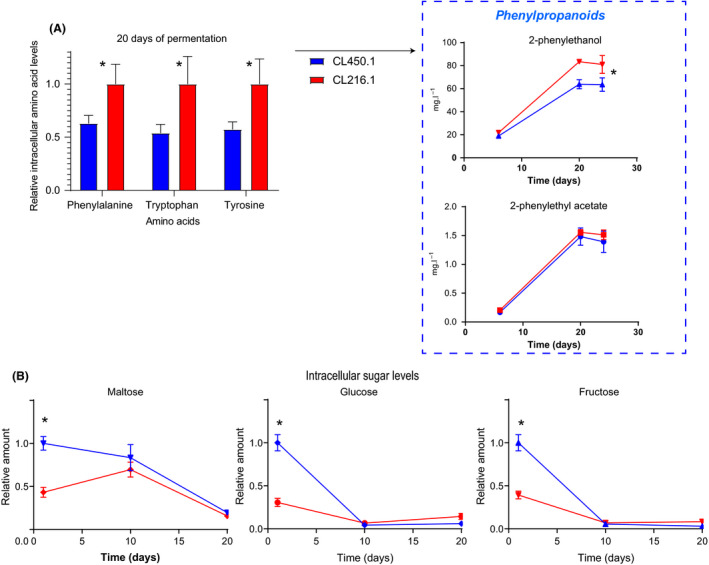
Intracellular metabolite analysis of native *S. eubayanus* strains. Intracellular metabolites in CL450.1 and 216.1 are shown in relative values for (A) amino acid phenylalanine, tryptophan and tyrosine together with phenylpropanoid levels derived from the amino acid metabolism. B. Maltose, glucose and fructose relative values after 20 h of fermentation are shown. Relative values were estimated based on the highest value throughout the whole fermentation sampled points. Fermentations were performed in quadruplicate (*n* = 4), and a one‐way ANOVA test was performed to evaluate for statistically significant differences between strains. (*) denotes time points exhibiting significant differences between strains.

We found significant differences between strains in intracellular metabolite levels throughout the fermentation process (Fig. 3). To examine how intracellular metabolite changes impacted fermentations dynamics, a metabolomic assay was performed across different fermentation stages. We measured 23 amino acids, eight carbon compounds and 14 organic acids at 24, 240 and 480 h of fermentation, and relative values were estimated per compound. Strains CL216.1 and CL450.1 showed remarkable differences for many metabolites (Table S4), indicating important differences in the production of secondary metabolites, nitrogen consumption and carbon sources. Clear increases in the intracellular levels of various by‐products of the pentose phosphate (erythritol) and chorismate (phenylalanine, tyrosine, and tryptophan) pathways were observed in the CL216.1 fermentation, especially after 240 h (Fig. 3A). This result suggests a higher activity of these pathways in the strain. The previous observations were notably in accordance with the higher extracellular phenylpropanoid levels, including 2‐phenylethanol and phenylethyl acetate, also reported in CL216.1 fermentations (Table S3C, Fig. 3A).

Strain CL216.1 accumulates higher levels of organic acids by the end of the fermentation, in contrast to CL450.1, which showed lower levels during the whole fermentative process. In this context, higher levels of the two main fatty acids involved in membrane bilayer composition of yeasts – stearic and palmitic acids – were obtained in CL216.1 strain (Table S4D). Interestingly, there was a clear increase in the level of both fatty acids from 240 to 480 h of fermentation, which coincides with the trend in activity of the pentose phosphate pathway (PPP) previously intuited in CL216.1. In terms of fermentable sugars, the CL450.1 strain accumulates a greater proportion of fructose, glucose and maltose during the early stages of the fermentation (*P‐*value < 0.05, ANOVA, Fig.3B) in contrast to CL216.1, which accumulates greater levels of most sugar by the end the fermentation (Table S4). After 240 h of fermentation, both strains CL216.1 and CL450.1 had, respectively, totally and almost totally consumed the first two preferential carbon sources, glucose and fructose present in wort, and started to take up the available maltose (see previous section). This represents an important transition in cell metabolism during fermentation.

Finally, intracellular amino acid content differed between strains (*P‐*value < 0.05, paired Student’s *t*‐test), where CL216.1 showed greater levels for most of the amino acids by the end of the fermentation. For example, we detected increasing intracellular levels of alanine and phenylalanine in CL216.1 during fermentation, likely impacting the production of acetaldehyde and 2‐phenylethanol respectively (Table [Link], [Link]). Altogether, the levels of each metabolite evaluated varied widely across the different strains in brewing conditions, with a higher proportion of intracellular nitrogen compounds and organic acids in CL216.1, likely due to a better fermentation performance in this genetic background.

### *De novo* genome assembly reveals widespread structural variants and gene repertoire diversification among *S. eubayanus* strains

**Figure 4:**
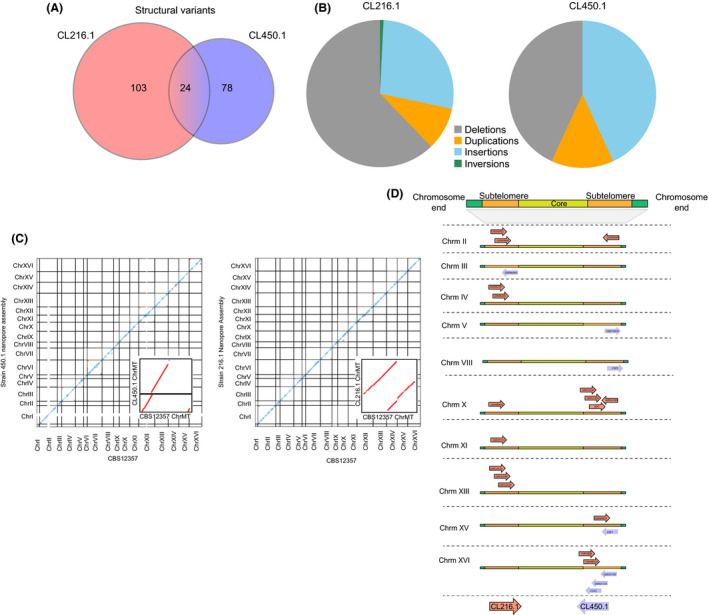
Identification of genomic rearrangements in *S. eubayanus* strains. A. Structural variants detected in both strains. Structural variants are defined as insertions, deletions, duplications and inversions above 50 bp. B. Pie chart of the SVs detected in CL216.1 (left panel) and CL450.1 (right panel). C. Dot plot representation of DNA sequence identity between CL450.1 (left panel) and CL216.1 (right panel) and the CBS12357 type strain and (D) chromosomal location of subtelomeric accessory genes found in *S. eubayanus.*

To obtain insights into the molecular and genetic differences between CL450.1 and CL216.1 strains, we re‐sequenced both genomes using Oxford Nanopore Technology with R9 flowcells. Reads for CL450.1 and CL216.1 were downsampled to 40× coverage, giving mean read lengths of 15.4 and 7.3 kb, prior to de novo assembly respectively. De novo genome assemblies gave 17 nuclear chromosomes, while CL216.1 contained a single collinear mitochondrial contig (chrMT) and CL450.1 contained two contigs not completely covering the reference chrMT. The quality of the genome assembly was evaluated using BUSCO. These results indicate that the genome completeness is 98.6 and 98.7 for CL216.1 and CL450.1 respectively. After polishing, the final annotated genomes predicted 5,508 and 5,523 ORFs for CL216.1 and CL450.1 respectively. Using whole‐genome alignment, both assemblies were compared with the reference genome and found a total of 127 and 102 structural variants (SVs) greater than 50bp in CL216.1 and CL450.1 respectively (Fig. 4A). Out of 205 unique SVs detected, 24 of them were common between both strains, demonstrating extensive genetic differences between isolates (Table S5D). Among all variants, deletions and insertions were highly enriched in both genomes, while inversions and duplications were less common (Fig. 4B). Furthermore, several genes involved in fermentation processes were found within these SVs, representing likely candidates for the observed differences in fermentation performance between strains. For example, two interesting deletions were found in CL216.1, one containing *PDR3* (gene encoding for a transcriptional activator of the pleiotropic drug resistance network and involved in diauxic shift) and another one *SNF3* (gene encoding for a hexose sensor, induces the glucose transporter’s expression) in CL216.1. Indeed, when we compared our genome assemblies for each strain with the CBS12357^T^ genome, we found perfect synteny, except for subtelomeres. Nevertheless, none of these represented translocations involving different chromosomes (Fig. 4C).

The GO term analysis on the complete genomes showed a very similar distribution pattern of ORFs in both genomes (Table S6A). Additionally, to determine the accessory ORFs on each strain likely responsible for specific phenotypic characteristics, we compared the set of predicted ORFs between both strains. Overall, we found 57 and 51 putative accessory ORFs (i.e. ORFs not shared between strains) in CL450.1 and CL216.1 strains, respectively (Table S6B–C), many of which were located in subtelomeric regions (Fig. 4D). The accessory ORFs found in CL216.1 were mainly related to catalytic activity, while molecular binding function was enriched in the set found in CL450.1 (Table S6B–C).

### Transcriptome analysis in beer wort conditions reveals different expression patterns

**Figure 5:**
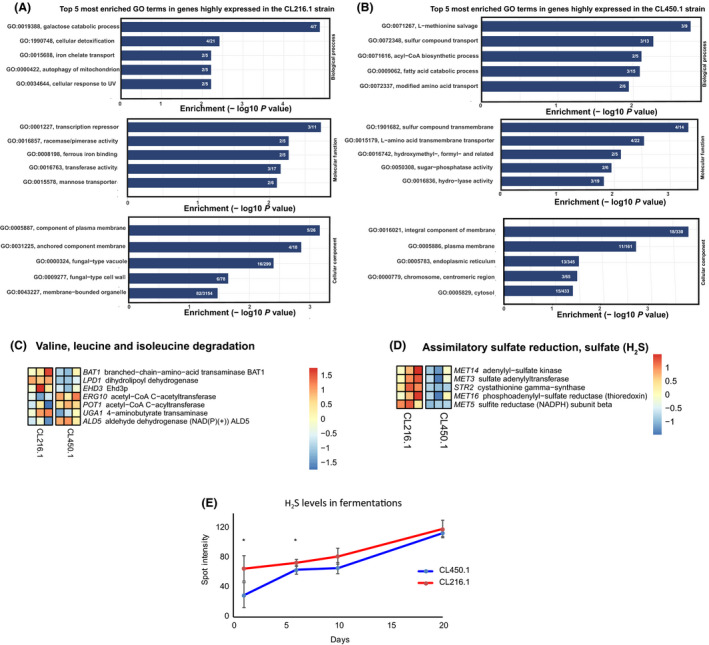
GO terms and KEGG enrichment analysis of differentially expressed ORFs between *S. eubayanus* strains in beer wort. A. Bar plots show the five most enriched GO terms in upregulated DEGs in CL216.1 and (B) CL450.1. C. Heatmap of ‘Valine, leucine and isoleucine degradation and (D) ‘Assimilatory sulfate reduction, sulfate (H_2_S)’ KEEG pathways in CL216.1 and CL450.1. Each column represents a replicate (E) H_2_S production during wort fermentation in 2L tanks.

To explore differences in global gene expression between the CL216.1 and CL450.1 strains during fermentation, we performed RNA‐seq analysis on samples collected 24 h after inoculation in wort. We first identified 1‐to‐1 ORFs that were correspondingly assembled, i.e. at least 96% of identity between orthologues. In this way, we compared expression levels for 4,934 genes and identified a total of 800 ORFs exhibiting significant expression differences between both genetic backgrounds (Table S7). GO term enrichment analysis of these genes revealed that these strains upregulated distinct metabolic pathways during early fermentation stages (Fig. 5A, Table S8A), in agreement with metabolome and volatile compound production differences. We found that genes involved in sugar metabolism were highly expressed in CL216.1 compared with CL450.1, among which ORFs related to galactose metabolism and hexose transport were found (Fig. 5A). In contrast, in CL450.1 we found enrichment related to amino acid metabolism and transport, such as L‐methionine salvage and sulfur compound transport, suggesting differences in H_2_S levels between strains (Fig. 5B).

Subsequently, to identify which differentially expressed ORFs were either up‐ or downregulated in different pathways, expression differences were mapped to the KEGG pathway database (Table S8B). Among the top enriched pathways, we found the ‘*Valine, leucine and isoleucine degradation’* exhibiting high expression levels in 5 out 13 ORFs within this pathway in CL450.1 (Fig. 5C). Similarly, ‘*Butanoate metabolism*’ and ‘*Assimilatory sulfate reduction, sulfate (H_2_S)*’ were found overexpressed in CL216.1, the latest including *MET* ORFs (Fig. 5D). Therefore, we estimated the relative amount of H_2_S formed throughout the fermentations using a silver nitrate‐based assay. Indeed, we found that CL216.1 produced significantly greater levels of H_2_S at the initial stages of the fermentation process (*P*‐value < 0.05, Student’s *t*‐test), yet this difference disappeared after 576 h of fermentation (Fig. 5E). Altogether, our results demonstrate that these pathways are directly involved in the production of volatile compounds explaining differences in aroma profiles between strains.

## Discussion

*Saccharomyces eubayanus* represents a new and promising production yeast for brewing, capable of providing novel flavours profiles to improve the organoleptic properties of lager beers (Gibson, *et al.*, 2017). However, thus far we know little about its biology and physiological behaviour under fermentative conditions, where only few isolates have been biologically characterized, hampering its wider utilization under industrial conditions. Specifically, only a half‐dozen isolates have been characterized for diverse brewing parameters, limiting our understanding of the species potential (Krogerus, *et al.*, 2015; Mertens, *et al.*, 2015). In this study, we used the natural *S. eubayanus* genetic variation as a tool to understand the influence of the brewing conditions on molecular dynamics and yeast biology, and their impact on the volatile compound profile of beers. Interestingly, isolates show wide differences in terms of fermentative capacity, with some isolates showing outstanding industrial potential (Libkind, *et al.*, 2011; Eizaguirre, *et al.*, 2018; Nespolo, *et al.*, 2019). Nevertheless, their fermentative capacity is still lower compared with that of other commercial lager strains, such as *S. pastorianus* W34/70 (Nespolo, *et al.*, 2019), generating the need to improve their fermentative capacities to make commercial application more feasible (Nespolo, *et al.*, 2019). Furthermore, in our study the CBS12357^T^ type strain showed a high fermentative capacity. This strain has been widely used in different studies as a representative strain of the *S. eubayanus* species, exhibiting an aroma profile towards volatile esters (Libkind, *et al.*, 2011; Krogerus et al., 2017a,2017b; Brickwedde, *et al.*, 2018; Eizaguirre, *et al.*, 2018). On the other hand, the P1C1 isolate is known to represent an admixed isolate from New Zealand belonging to the PB lineage; however, its fermentative capacity has not been studied in depth (Gayevskiy and Goddard, Langdon, *et al.*, 2019; Nespolo, *et al.*, 2019). In general, all the strains used in this study can efficiently grow in beer wort (fresh mass), suggesting that the increased ethanol production is due to maltose uptake improvements that increase fermentative capacity (Goold, *et al.*, 2017). Maltotriose consumption was not observed in any of the strains evaluated in our study, in agreement with previous reports (Baker and Hittinger, 2019; Brouwers, *et al.*, 2019). As a result of domestication, the majority of baking and brewing *S. cerevisiae* strains are able to consume maltotriose (Goncalves, *et al.*, 2016; Baker and Hittinger, 2019), while wild *Saccharomyces* strains are incapable of its utilization. To understand the capacity of the brewing hybrid to use maltotriose, a recent study demonstrated the transcriptional cross‐talk between hybrid *S.cerevisiae* x *S.eubayanus* sub‐genomes, where the Holarctic *S. eubayanus* (the closest known *S. eubayanus* relative of the lager yeast) maltose‐hydrolase and maltose‐transporter genes can be activated by maltose *S. cerevisiae* transcription factors, demonstrating how these hybrids were domesticated for centuries under brewing conditions (Brouwers, *et al.*, 2019).

Interestingly, some strains showed a greater tendency to produce higher alcohol levels (i.e. CL450.1 and P1C1), while others tended towards ethyl ester production (i.e. CL216.1 and the CBS12357^T^ type strain). Therefore, the aroma profiles obtained in our study demonstrate that Patagonian isolates produce significantly different concentrations of a series of volatile compounds normally found in beer. This suggests that differences in the strains’ genetic backgrounds could be exploited to produce a range of novel beers (Olaniran, *et al.*, 2017). In this context, no clear correlation between volatile compound production and genetic clustering or geographic origin was obtained, likely suggesting that population structure does not determine the observed patterns. Furthermore, compound differences can be found across the whole fermentation process, demonstrating how dynamic the process is (Steensels, *et al.*, 2014). Other studies have revealed high levels of ethyl decanoate and isobutanol in *S. eubayanus* strains from Argentina, which can be inherited in *S. cerevisiae* x *S. eubayanus* hybrids (Mertens, *et al.*, 2015; Krogerus et al., 2017a,2017b). Differences in volatile compound production may be due to amino acid utilization (Olaniran, *et al.*, 2017; Holt, *et al.*, 2018). During the fermentation process, yeasts assimilate amino acids and other nutrients in order to proliferate and produce biomass. Simultaneously, different volatile compounds are produced, such as esters and higher alcohols, together with undesired compounds, like phenolic off‐flavours and sulfur compounds (Kobayashi, *et al.*, 2006; Procopio, *et al.*, 2011; Holt, *et al.*, 2018). Indeed, the metabolome analysis shows unique patterns between strains, where we observed remarkable differences in the nitrogen and carbon sources consume/accumulation ratios. Differences in amino acid accumulation could explain those differences found in the production of volatile compound and higher alcohols during the fermentation of CL216.1 and CL450.1 strains. The initial accumulation of isoleucine and later transformation by transamination, decarboxylation and reduction to 2‐methyl butanol in CL450.1 could explain the greater production of this compound compared with CL216.1 in the first stages of fermentation process. Similarly, the initial accumulation and subsequent depletion of phenylalanine in CL450.1 could correlate with greater 2‐phenylethanol levels by the end of the fermentation process. Transcriptome profiling also highlighted several pathways, such as ‘*valine, leucine and isoleucine degradation*’ highly expressed in CL450.1 that could likely impact sensory profiles. Indeed, these amino acids represent precursors of volatile compounds, such as undesirable butter‐tasting vicinal diketone diacetyl and 2,3‐pentanedione (Krogerus and Gibson, 2013), but also of 2‐methylpropanol and 3‐methylbutanol (Eder, *et al.*, 2018). On the other hand, CL216.1 is able to overexpress the ‘*Assimilatory sulfate reduction (H_2_S)’* pathway, which correlates with greater *H_2_S* levels found in this strain during brewing. Still, CL216.1 produces greater levels of flavour‐active esters, such as ethyl octanoate and ethyl acetate, likely providing a fruity sensation to beer. Volatile esters are of major industrial interest since their presence in beer and wine determines the fruity aroma of these beverages, even at low concentrations (Verstrepen, *et al.*, 2003; Saerens, *et al.*, 2008; Olaniran, *et al.*, 2017).

In conclusion, we observed different volatile compound profiles between *S. eubayanus* strains that included higher alcohols and volatile esters, likely influenced by differences in uptake and the accumulation of precursor amino acids. In the two strains studied in simulated industrial conditions, CL216.1 showed a better fermentation profile and the production of greater levels of volatile esters. These differences can be explained by the differential expression of ORFs involved in both hexose transport and amino acid metabolic and transport processes together with acyl‐CoA and carbohydrate metabolic processes, culminating in varying intracellular levels of amino acids and sugars. Our findings provide new insights into the molecular pathways induced in different *S. eubayanus* genetic backgrounds that underlie unique volatile compound profiles in brewing conditions and provides a good starting point for further research to select new strains for the beer industry.

## Experimental procedures

### Yeast strains

The strains utilized in this study are listed in Table S1 and were collected from Chilean localities of Villarrica (S39°16′00″ O72°13′00″) and Coyhaique (S45°33'14" O71°59'28.72") during the South Hemisphere Summer in 2017 (Nespolo, *et al.*, 2019). All strains are available upon request.

### Fermentations in beer wort

Fermentations were carried out in at least two biological replicates depending on the experiment. Fermentations were conducted using a 15 °Plato (°P) wort at 15°C in either 50 ml (micro‐fermentations) or two litre‐scale fermentations. That being said, °P refers to Degrees Plato and corresponds to a measurement of the concentration of dissolved solids in a brewery wort, mainly sugars derived from malt but also including other soluble material in wort, as a percentage by weight. The 15 °Plato wort (57.6 g maltose, 19.8 g maltotriose, 16.1 g glucose and 5.4 g fructose per litre) was produced at the VTT Pilot Brewery from barley malt. The worts were oxygenated to 15 mg L^−1^ prior to pitching (Oxygen Indicator Model 26073 and Sensor 21158; Orbisphere Laboratories, Geneva, Switzerland). Fresh mass of yeast was determined by centrifuging the cultures (9000 *g* for 10 min) in pre‐weighed centrifuge tubes and washing cell pellets with Milli‐Q water. This step was repeated twice, and any residual water was removed by pipette before calculation of mass. For the micro‐fermentations, the strains were initially grown with constant agitation in 50 ml of wort for 48 h at 15°C. Following this, 50 ml of fresh wort was inoculated to a final concentration of 15 × 10^6^ viable cells ml^−1^ and fermentations were maintained for 168 h at 15°C.

Similarly, the 2 litre‐scale wort fermentations were carried out as previously described (Krogerus et al., 2017a,2017b). Briefly, fermentations were carried out in quadruplicate in two litre cylindroconical stainless steel fermenting vessels, containing two litres of wort medium. A 2 l 24 h pre‐culture was generated and used to inoculate 15 × 10^6^ viable cells ml^−1^. The fermentations were maintained at 15°C until alcohol levels did not increase more than 10% per day.

### HPLC and volatile compound quantification

At the end of the fermentation, the fermented wort was centrifuged at 9000 *g* for 10 min and the supernatant collected. From this, sugar content was determined by HPLC using a Waters 2695 separation module and Waters system interphase module liquid chromatograph coupled with a Waters 2414 differential refractometer (Waters Co., Milford, MA, USA). A Rezex RFQ‐Fast Acid H^+ ^(8%) LC column (100 x 7.8 mm; Phenomenex, Torrance, CA, USA) was equilibrated with 5 mM H_2_SO_4_ (Titrisol, Merck, Germany) in water at 80°C, and samples were eluted with 5 mM H_2_SO_4_ in water at a 0.8 ml min^−1^ flow rate. The alcohol level (% vol/vol) of samples was determined from the centrifuged and degassed fermentation samples using an Anton Paar density meter DMA 5000 M with Alcolyzer beer ME and pH ME modules (Anton Paar GmbH, Graz, Austria).

Volatile compounds, such as acetaldehyde, higher alcohols and esters, were determined by headspace gas chromatography with flame ionization detector (HS‐GC‐FID) analysis. Four millilitre samples were filtered (0.45 µm pore size) and incubated at 60°C for 30 min, and then 1 ml of gas phase was injected (split mode, 225°C, split flow of 30 ml min^−1^) into a gas chromatograph equipped with an FID detector and headspace autosampler (Agilent 7890 series; Agilent, Santa Clara, CA, USA). Analytes were separated on a HP‐5 capillary column (50 m by 320 µm by 1.05 µm column; Agilent). The carrier gas was helium (constant flow of 1.4 ml min^−1^). The temperature program was 50°C for 3 min, 10°C per min to 100°C, 5°C per min to 140°C, 15°C per min to 260°C, and then isothermal for 1 min. Compounds were identified by comparison with authentic standards and were quantified using standard curves. 1‐Butanol was used as an internal standard.

Relative H_2_S concentrations were estimated using a microplate‐based silver nitrate assay as described by Duan et al. (Duan, *et al.*, 2004). In brief, 200 µL samples drawn from the wort fermentations were placed in a 96‐well plate. The plate was then overlaid with a membrane (3 MM chromatography paper, GE Healthcare, Chicago, IL, USA) freshly impregnated with a 20% silver nitrate solution. The plate was finally sealed with a Breathe‐Easy® sealing membrane (Sigma‐Aldrich, St Louis, MO, USA) and wrapped in aluminium foil. The plate was incubated for 24 h at 25°C, after which the membrane was carefully removed. The membrane was scanned with a Bio‐Rad GS‐710 densitometer, and the intensity of the dark spots (formed from precipitated silver sulphide) was quantified with the supplied Image Lab software (Bio‐Rad, Hercules, CA, USA).

### Metabolome assay

Intracellular metabolites were extracted using a modified pure cold methanol extraction protocol adapted from Villas‐Bôas, *et al.* (2005). The cell pellet was collected, and a 250 μl volume of cold absolute methanol (−40ºC) was added. The sample was mixed and rapidly transferred into dry ice for 30 min. Later, the sample was defrosted in an ice bath during 10 min, and posteriorly, the cells were centrifuged at 4000 rpm for 10 min at 0ºC. The first supernatant was transferred to a tube, and the extracted pellet was newly washed with another 250 μl volume of pure cold methanol. The second extraction was performed to ensure the obtention of any metabolites left after the first extraction. The two extractions were joint and stored at −80ºC for the subsequent GC‐MS analysis. In order to quantify the metabolites, an extract volume corresponding to 0.5 mg of yeast fresh weight (2–5 μl) was mixed with 3 μl of internal standard (0.2 mg ml^−1^ ribitol in water) and dried in a SpeedVac. The derivatization was carried out resuspending the dried sample in 40 μl of 20 mg ml^−1^ methoxyamine hydrochloride in pyridine and posteriorly was incubated for 90 min at 37ºC, followed by addition of 60 μl MSTFA (*N*‐methyl‐*N*‐[trimethylsilyl]trifluoroacetamide) and 6 μl of a retention time standard mixture [3.7% (w/v) mix of fatty acid methyl esters spanning from 8 to 24°C] and posterior incubation at 37ºC during 30 min. Two microlitres of sample was injected in the split and split‐less mode for a better metabolite detection range in a 6890 N gas chromatograph (Agilent, USA) coupled to a Pegasus 4D TOF mass spectrometer (LECO, St Joseph, MI, USA). Gas chromatography was carried out on a BPX35 (30 m × 0.32 mm × 0.25 μm) column (SGE Analytical Science Pty, Australia) with helium as a carrier and a constant flow of 2 ml min^−1^. The liner was set at 230°C. Oven program was 85°C for 2 min, 8°C min^−1^ ramp until 360°C. Mass spectra were collected at 6.25 spectra s − 1 in the m/z range 35–900 and ionization energy of 70 eV. Chromatograms and mass spectra were evaluated by mean CHROMATOF program (LECO).

### Genome sequencing and annotation

The genomic DNA was extracted using Qiagen Genomic tip 20/G and both repaired and dA‐tailed using FFPE (NEBnext) and UltraII end‐prep (NEBNext) kits respectively. EXP‐NBD103 barcodes and SQK‐LSK108 adapters were ligated, and library was loaded on R9 flowcells (Nanopore, Oxford, UK). Raw fast5 files were converted for fastq files and debarcoded using Guppy 2.3.5 (Ueno, *et al.*, 2003). Barcode and adapter sequences were trimmed using Porechop (https://github.com/rrwick/Porechop) and downfiltered to 40× coverage using Filtlong (https://github.com/rrwick/Filtlong). Canu v1.8. (https://github.com/marbl/canu) with default setting was used for assembly, followed by two rounds of both nanopolish (Loman, *et al.*, 2015) and pilon (Walker, *et al.*, 2014). The completeness of the genome assembly was evaluated using BUSCO (Simao, *et al.*, 2015).

The genome assemblies were annotated using the pipeline LRSDAY (Yue and Liti, 2018) using the complete genome of the CBS12357^T^ type strain as training input for AUGUSTUS (Stanke and Morgenstern, 2005) and additionally assisted with the respective transcriptome assembly generated by TRINITY (Grabherr, *et al.*, 2011). Gene function was predicted using as reference the *S. cerevisiae* S288C genome, and the GO term enrichments were evaluated using Blast2GO and TopGO (Alexa, *et al.*, 2006). All the other parameters were set up as default. Assemblies were compared with CBS12357^T^ using nucmer (Marçais, et al. 2018), and structural variants (SVs) called using MUM&Co (https://github.com/SAMtoBAM/MUMandCo). SVs between both strains were considered the same event if the SV type was identical and both the beginning and end of the SVs were within 50 bp. Reads are available in the Biosample Database Project # PRJNA578372.

### RNA‐sequencing and differential expression analysis

The two strains, CL216.1 and CL450.1, were fermented in 2 l wort tanks. Fermentations were carried out as previously described in triplicates for each individual for 24 h. Cultures were harvested using a 5 ml needle, and cells were collected by centrifugation. Cells were then treated with 2 U of Zymolyase for 30 min at 37°C. RNA extraction was performed utilizing the E.Z.N.A. Total RNA Kit I (OMEGA BIO‐TEK, Norcross, GA, USA) according to the supplier’s instructions. RNA samples were then treated with DNase I (Promega, Madison, WI, USA) to remove genomic DNA traces, and total RNA was recovered using the GeneJET RNA Cleanup and Concentration Micro Kit (Thermo Scientific, Waltham, MA, USA). RNA integrity was confirmed using a Fragment Analyzer (Agilent). The RNA‐seq libraries were constructed using the TruSeq RNA Sample Prep Kit v2 (Illumina, San Diego, CA, USA). The sequencing was conducted using paired‐end 100 bp reads on an Illumina HiSeq X Ten in a single lane for the six samples. Reads are available in the Biosample Database Project # PRJNA578372.

The quality of the sequencing reads was assessed using fastqc (http://www.bioinformatics.babraham.ac.uk/projects/fastqc/). Reads were processed using fastp (‐3 ‐l 40) (Chen, *et al.*, 2018). Reads were mapped to their own transcriptome obtained with the annotation of the long‐read assemblies using RNAstar ver. 2.7.0 (Dobin, *et al.*, 2013). To obtain the set of 1‐to‐1 orthologous genes, we performed a reciprocal BLASTN against the two transcriptomes. Genes were considered orthologues if they showed at least 96% of identity and the blast alignment covered at least 96% of both transcripts complete sequence. Statistical differential expression was analysed using DESeq2 package in r (Love, *et al.*, 2014). Genes showing an adjusted *P*‐value of 0.05 or less were considered as differentially expressed genes (DEGs). Analysis of GO term enrichment was done with the R package TopGO (Alexa, *et al.*, 2006). KEGG pathway enrichment of DEGs was analysed using the Metascape website (Zhou, *et al.*, 2019).

### Statistical analysis

Differences in metabolite levels, physicochemical parameters and volatile compounds were compared using a one‐way ANOVA test. The comparison of volatile compound concentrations during the different fermentation stages was compared between strains using a paired *t*‐test. All analyses were performed using GraphPad Prism (version 6.01, 2012). *P* values < 0.05 were considered as statistically significant.

## Conflicts of interest

None declared.

## Supporting information


**Table S1**. Strains fermentation parameters after 168 hours of fermentation. Sugar levels are informed in terms of consumption levels. Ethanol and glycerol levels correspond to the produced amount during fermentation, while wort values correspond to available g l^‐1^ before fermentation. R1 and R2 refer to biological replicates.

**Table S2**. Volatile compound production (mg l^‐1^) in *S. eubayanus* strains. Replicates are indicated as R1 and R2.

**Table S3**. Sugar consumption and Volatile compound production in 2 l tanks for CL450.1 and CL216.1 strains after 144, 480 and 576 hours of fermentation. A one‐way ANOVA test was performed to evaluate for statistically significant differences between strains.

**Table S4**. Relative Intracellular amino acids. Relative values to the highest level on each metabolite are informed. A one‐way ANOVA test was performed to evaluate for statistically significant differences between strains.

**Table S5**. General statistics of the genome assemblies in CL216.1 and CL450.1.

**Table S6**. ORFs mapped in CL216.1 and CL450.1.

**Table S7**. RNA‐seq results.

**Table S8**. GO term and KEEG enrichment.
